# Genetics, Safety, Cost-Effectiveness, and Accessibility of Injectable Lipid-Lowering Agents: A Narrative Review

**DOI:** 10.1155/2023/2025490

**Published:** 2023-03-08

**Authors:** Abdulmajeed Abdulghani A. Sindi

**Affiliations:** Department of Basic Medical Sciences, Faculty of Applied Medical Sciences, Al-Baha University, Al-Aqiq, Albaha, Saudi Arabia 65779-7738

## Abstract

Cardiovascular disease causes significant personal, financial, and societal burden and is a major cause of mortality and morbidity globally. Dyslipidemia has proven to be a major factor that contributes to its increased incidence; thus, since a long time, low-density lipoprotein cholesterol-lowering therapies have been employed to reduce coronary artery disease-associated mortality. The first-line therapy for hyperlipidemia and dyslipidemia is statins. Evidence showed that statins decrease the level of LDL-C resulting in a lower risk of CVD (20-25% for every decrease of 1 mmol/L). However, due to statin intolerance in some patients and despite using maximal doses, they have not been successful in lowering cardiovascular-associated mortality. Moreover, bococizumab was recently suspended due to its higher immunogenicity with time, resulting in less efficacy with long-term use. Alternatives to statins are PCSK9 inhibitors which are administered subcutaneously every two or four weeks. They are injectables with considerable lipid-lowering properties. This narrative review discusses their genetics, safety, tolerability, and cost-effectiveness. It also quantifies their benefit in certain subgroups by analyzing the findings from recent randomized clinical trials. Current data from phase 2 and 3 trials (ORION, ODYSSEY, and FOURIER) suggest a favorable profile for evolocumab, alirocumab, and inclisiran with minimal tolerable side effects and superior efficacy in statin-intolerant patients. Their cost-effectiveness has not yet been established clearly, but future outcomes seem promising.

## 1. Introduction

Cardiovascular diseases (CVD) were reported as the largest cause of death and disability in developing countries, taking an estimated 17.8 million lives worldwide with 330 million years of life lost and another 35.6 million years living with disability. According to the US Centers for Disease Control and Prevention, almost 610 thousand deaths occur yearly due to heart disease [1, 2]. In addition, it was reported that the rate of coronary artery disease (CAD) is increased by dyslipidemia [3]. Heterozygous familial hypercholesterolemia (FH), which is a hereditary disease characterized by increasing low-density lipoprotein cholesterol (LDL) levels from birth, exposes arteries to high levels of atherogenic lipoproteins in early life and was found associated with higher risk of cardiovascular events. Dyslipidemia can be clinically defined as elevated total cholesterol or LDL-C levels and reduced HDL-C (high-density lipoprotein) levels. Low HDL cholesterol and high TG concentrations have been implicated as other possible independent predictors of CVD [4], and the combination of these two conditions is called as atherogenic dyslipidemia.

To counter the atherosclerotic cardiovascular disease risk, new lipid therapies such as statin therapy have flourished that decrease atherogenic particles like LDL and Lp(a) (lipoprotein(a)) and triglyceride (TG)-rich VLDL (very-low-density lipoprotein) and remnants [4]. Systematic reviews document a significant role that reduction in LDL-C levels has on the decrease in cardiovascular risk [5]. Yet, patients at very high risk of cardiovascular disease must maintain LDL-C levels as low as 70 mg/dL and less as recommended by the guidelines. Statins' role is mainly reducing the synthesis of cholesterol by blocking 3-hydroxy-3-methylglutaryl-CoA (HMG-CoA) reductase. However, it is reported that even when high and very high cardiovascular risk patients use this treatment with lifestyle modification, they stay beyond the treatment targets with high risk of developing cardiovascular events such as stroke, myocardial infarction, revascularization, and even death [6]. Given the high prevalence of patients at high cardiovascular risk with no progress even with a maximized statin treatment dose, an alternative more intensive cholesterol-lowering therapy is needed [7].

Injectable alternatives to statins such as proprotein convertase subtilisin/kexin type 9 (PCSK9) inhibitors contribute to the lowering of lipid levels. The 692 amino acid of the secreted protease PCSK9, which is arranged in three domains (prodomain, catalytic domain, and C-terminal domain), regulates the LDL receptor (LDL-R). It is bound to LDL-R to form a LDL-R–PCSK9 complex. This complex is targeted for lysosomal degradation in hepatocytes. In the presence of a high concentration of PCSK9, this interaction results in a lower number of LDL-R on the hepatocyte cell surface and consequently increases the concentration of circulatory LDL [8]. On the other hand, multiple PCSK9 inhibitors have been proven to be effective with a variety of inhibition mechanisms. The mechanisms include blocking LDL receptor binding, preventing the formation of the LDL-R-PCSK9 complex that leads to an increased number of LDL-R migration to the cell surface for circulating LDL removal or inhibiting the synthesis of PCSK9 at the translation level by interrupting the PCSK9 gene expression, and lastly blocking the process of autocatalysis and consequently blocking the maturation of PCSK9 and cell secretion [9, 10]. In this review, it is aimed at focusing on the injectable alternatives to statins, PCSK9 inhibitors, that contribute to the lowering of lipid levels under four main themes: genetics, safety profile, cost effectiveness, and accessibility. In addition, it is also aimed at applying a holistic approach for assessing the feasibility of lipid-lowering injectables, alirocumab (Praluent), evolocumab (Repatha), and siRNA therapy targeting PCSK9 (inclisiran), by discussing the findings of FOURIER, ORION, open-label OSLER, and ODYSSEY trials, and eventually summarizing their impact in lowering cardiovascular-associated mortality.

## 2. Methodology

### 2.1. Search Strategy

Systematic literature searches were performed in the following databases until December 2022: PubMed, Embase, Medline, the Cochrane Central Register of Controlled Trial (CENTRAL), and ClinicalTrials.gov. We used different combinations of the following search terms: “Injectables,” “metformin,” “Lipid Lowering,” and “PCSK9 inhibitors”. Relevant citations were screened by reading the titles and abstracts of the citations in the results without language, ethnicity, or regional restrictions. Animal studies, case reports/series, opinions, pieces, editorials, and commentaries were excluded.

### 2.2. Outcomes

Outcomes of interest were the genetic basis of response to injectable lipid-lowering agents, the safety and adverse effects of these agents, the cost-effectiveness of injectable lipid-lowering agents, and their accessibility and availability to patients.

### 2.3. Types of Participants

The population of interest was adult patients with elevated lipid levels or diagnosed with dyslipidemia who were prescribed injectable lipid-lowering agents.

### 2.4. Type of Studies

The literature review included both randomized controlled trials and observational studies including cohort, descriptive, cross-sectional, and case-control studies.

### 2.5. Quality Assessment

Finally, 103 relevant citations published between 2013 and 2022 were included, which had good strength of evidence.

## 3. PCSK9 Antibody Therapy

High LDL-C concentrations are reasoned for some genetic polymorphisms and not due to lifestyle changes. A genetic sequence of PCSK9 protein was revealed in the early 2000s, and studies showed that PCSK9 mutation is implicated in cholesterol metabolism. Subsequently, two dominant heterozygous missense mutations of PCSK9, p.S127R and p.F216L, were identified through DNA sequencing in three French families with hypercholesterolemia [11]. Therefore, PCSK9 was accounted as the third gene responsible of familial hypercholesterolemia that required further bimolecular research to fully understand its association with LDL receptors. The two-point mutations in the *PCSK9* gene identified in French families of FH had a leading role in LDL-C metabolism with no mutations in other FH-related genes. Additional mutation in PCSK (p.D374Y) was also identified to be correlated with a high level of circulating LDL-C in a Utah and Norwegian pedigree. PCSK9 is synthesized by hepatocytes and forms a complex with LDL-R on the surface of hepatocytes, resulting in LDL-R internalization and deterioration, which in turn increases the LDL-C blood level. Initially, therapeutic approaches to lower circulating levels of PCSK9 have focused on the use of monoclonal antibodies that sequester PCSK9 in the reticuloendothelial system, preventing it to bind to the LDL receptor [12]. Preclinical studies included PCSK9-knockout mice, which have almost 2.8 times increased LDL receptor expression and almost 50% lower total plasma cholesterol and lower apoB concentrations compared to wild-type mice. In this experiment, LDL-C is lower by 80% than the control group. In addition, PCSK9 deletion in mice lowers the occurrence of atherosclerosis according to LDL-R. Based on these results, efforts were put to develop targeted therapies [13]. Monoclonal antibodies are effective in inhibiting PCSK9 and reducing LDL levels. Recently, six antibodies are tested and developed such as alirocumab, evolocumab, and bococizumab.

In a 10-year period, two meta-analyses of clinical trials comparing anti-PCSK9 treatment with placebo or ezetimibe (a blocker for the absorption of cholesterol agent) were published [14, 15]. This includes over 10,000 hypercholesterolemia subjects. Alirocumab and evolocumab are the most known PCSK9-inhibiting agents with approval by the US Food and Drug Administration (FDA) in patients with high cardiovascular risk and need beyond standard lipid-lowering therapy. In addition, patients with familial homozygote and primary hypercholesterolemia are also approved to be treated with evolocumab. These two are fully human IgG subtypes that bind in a 1 : 1 stoichiometry to circulating PCSK9, inhibiting the PCSK9-LDL receptor bind [16]. Alirocumab or evolocumab are injected subcutaneously (75-150 mg and 140-420 mg, respectively) resulting in an excess of antibodies that in turn can pick up both circulating PCSK9 (after a couple of hours of administration) and newly secreted PCSK9 (after a couple of days of administration). Individuals under targeted monoclonal antibodies were found to have 10-fold and even 20-fold in some cases of plasma concentrations of PCSK9 [17].

The Global Assessment of Plaque Regression with a PCSK9 Antibody as measured by Intravascular Ultrasound trial, known as the GLAGOV trial, investigated the effect of PCSK9 inhibition on atherosclerosis. They reported that the decrease in LDL-C due to the administration of evolocumab to statin treatment resulted in atheroma regression and helped in lowering the atherosclerotic cardiovascular risk [18]. Indeed, another trial known as FOURIER trial was conducted on a large sample of patients with previous atherosclerotic cardiovascular disease (ASCVD) and on maximum tolerated doses of statin therapy (in whom 2/3 were administrated with high-intensity statin), yet these patients still had an LDL − C ≥ 70 mg/dL or a non-HDL cholesterol ≥ 100 mg/dL [19]. In this trial, the two comparative groups received randomly either subcutaneous injections of evolocumab or placebo. Findings showed that the treatment group had 59% lower LDL-C (92 mg/dL vs. 30 mg/dL) and the outcome including 2-year cardiovascular death, MI, or stroke was significantly lower by 20% (7.4% vs. 5.9%) [19]. However, a minor number of patients treated in the FOURIER trial exhibited a nonresponse to PCSK9 inhibitor [19]. In the diagnosis of nonresponse to PCSK9 inhibitor, the increased levels of PCSK9 inhibitors in the plasma of some patients may indicate a resistance of PCSK9 inhibitors due to different reasons such as PCSK9 dysfunctional mutation. On the other hand, another diagnosis of nonresponse to PCSK9 inhibitors with unelevated levels of PCSK9 inhibitor in the plasma was suggested with either due to lack of antibody adherence or disposition, inappropriate injection technique or dermatological problems to prevent monoclonal antibody pass into the circulation, mutation in Ab-PCSK9 recognition site, or internal production antibody against the injected monoclonal antibody [17].

The ODYSSEY Outcomes trial reported 15% lower cardiovascular events and deaths after alirocumab treatment in patients with acute coronary syndrome and higher-than-ideal atherogenic lipoprotein levels with maximally tolerated doses of statin therapy. ODYSSEY Outcomes is the second outcome trial with a PCSK9 inhibitor to show a reduction in LDL-C and cardiovascular endpoints [20]. Although the reduction of cardiovascular events and death was 5% less than the use of evolocumab in the FOURIER trial, this trial had different designs and settings than FOURIER, with the higher-risk group of patients enrolled and followed up for a longer period and having different dosing strategies and lastly evaluating a slightly different primary endpoint. According to the trial's findings, patients who have experienced an acute coronary syndrome (ACS) episode within the previous 1–12 months can dramatically reduce ischemic events, including all-cause mortality and MI, by taking alirocumab every other week. With alirocumab, the first and overall nonfatal incidents were decreased. Nearly 90% of these individuals were taking a powerful statin at a high dosage (atorvastatin or rosuvastatin). Early LDL-C reductions of more than 50% were noted, and they seemed to be more or less sustained during follow-up. Similar to ODYSSEY, Open-Label Study of Long-term Evaluation Against LDL-C (OSLER-1) clinical trial evaluated the durability of long-term efficacy and safety during treatment with evolocumab in hypercholesterolemia and showed a sustained reduction in LDL-C levels [21].

Although the main role of PCSK9 antibody therapy is involved in increasing LDL-R level and decreasing LDL-C levels, some PCSK9 inhibitors are also capable of lowering the concentrations of apolipoprotein B (apoB) and very-low-density lipoprotein (VLDL) cholesterol and in contrast slightly augmenting the concentrations of apolipoprotein A-I (apoA-I) and high-density lipoprotein cholesterol (HDL-C). This might be due to the fact that when the concentration of apoB decreases, it may cause the decrease of cholesterol level that is transferred from HDL to particles containing apoB [21]. PCSK9 inhibitors have an additional benefit when compared to statin therapy, in which they decrease lipoprotein a (Lp(a)) concentrations that are directly associated with a higher risk of cardiovascular diseases. The ODYSSEY trial was one of the first studies that demonstrated a therapeutic benefit with a decrease in Lp(a) that is unrelated to LDL-C. For patients with high baseline Lp(a) levels, this was especially significant [20]. This could be a brand-new treatment target for ACS sufferers. The lowering process of Lp(a) is believed to be that Lp(a) competes poorly with LDL for LDL-R binding. Thus, when PCSK9 inhibitors increase LDL-R expression and particularly when LDL levels are low, Lp(a) decreases. Other possible reason includes apoB or Lp(a) synthesis [22].

## 4. Small Interfering RNA-PCSK9 Targeted Therapy

An alternative to the use of monoclonal antibodies is through the administration of antisense siRNA molecules. Inclisiran (ALN-PCSsc) is a long-acting synthetic siRNA directed against the translation of PCSK9 protein. This siRNA is conjugated to triantennary N-acetylgalactosamine carbohydrates, which bind to abundant liver-expressed asialoglycoprotein receptors leading to a rapid uptake of inclisiran specifically into hepatocytes after subcutaneous injection. Its effect lasts longer than any other lipid-lowering drug agents [23]. Inclisiran roles may differ from monoclonal antibodies in which it does block the PCSK9 protein synthesis.

The analysis of the ORION-1 trial showed that when inclisiran was administrated in ASCVD patients or those at risk combined with high LDL-C, they had more significancy in lower LDL-C compared with the maximally tolerated doses of LDL-C-lowering therapy [22]. The ORION-9 phase 3 trials evaluated the administration of inclisiran sodium compared to placebo in 482 adults suffering from heterozygous familial hypercholesterolemia and who were on the maximum tolerated dose of statin therapy. The results showed a between-group difference of -47.9 percentage points of LDL cholesterol level (a reduction of 39.7% in the inclisiran group vs. an increase of 8.2% in placebo) whereas the time change in the LDL cholesterol level difference was -44.3% points (a reduction of 38.1% in the inclisiran group vs. an increase of 6.2% in the placebo group). Inclisiran was reported to reduce PCSK9 plasma concentrations (as high as 80%) and reduce total cholesterol, non-high-density lipoprotein cholesterol (non-HDL-C), apolipoprotein B, triglycerides, and Lp(a) levels (18.6 to 25.6%) [24].

However, these ORION trials proved the effect, like other monoclonal antibodies, on the lowering of LDL-C levels in addition to other lipids and lipoproteins, yet it is reported that PCSK9 plays a role in lipid and lipoprotein metabolism through an intracellular mechanism. Nishikido and Ray analyzed PCSK9 inhibitors as well as the outcomes of inclisiran phase I and II clinical trials. LDL receptor degradation is accelerated by plasma PCSK9, which causes a buildup of LDL-C in the bloodstream. Monoclonal antibodies are now used to sequester circulating PCSK9; however, these methods demand regular injections. With a rare dosing interval of twice a year, inclisiran offers advantages over monoclonal antibodies by reducing LDL-C by more than 50% while inhibiting the translation of PCSK9 mRNA and, as a result, turning off PCSK9 production [25]. The long-term safety of inclisiran in patients with high cardiovascular risk and elevated LDL-C is under investigation by a number of ongoing trials. According to a recent systematic review and pooled analysis of 5 RCTs (4226 participants), inclisiran has demonstrated positive impacts on serum lipid levels and a safety profile that is deemed acceptable [26].

## 5. Anti-PCSK9 Vaccines

Recent investigations with positive findings showed the efficacy of peptide-based vaccines in inhibiting PCSK9 protease to bind to LDL receptors or in degrading PCSK9 [27, 28]. For example, the AT04A vaccine reported a steady decrease in PCSK9 concentrations of almost 50% over a period of 12 weeks with also a reduction in atherosclerotic lesions and vascular inflammation and inflammatory biomarkers [29]. In animal models, peptide-based anti-PCSK9 vaccinations cause the production of antibodies that are long-lasting, highly affine, and useful for up to a year. PCSK9 is a very alluring target for LDL-C control due to its capacity to adjust circulating LDL-C concentrations. As a result, the peptide-based anti-PCSK9 vaccine represents a ground-breaking method and an effective tactic for the long-term control of LDL-C levels [28]. Another developed vaccine is the L-IFPTA+ formed by the junction immunogenic peptide with the surface of negatively charged nanoliposomes along with the adjuvant Alhydrogel®. The study showed that after twice injection of L-IFPTA, PCSK9 levels were significantly lower by 58.5% in C57BL/6 mice. After 8 weeks, LDL-C and TC levels showed a 51.7% and 44.7% decrease compared to controls [28].

## 6. Safety of PCSK9 Inhibitors on Neurocognitive and Other Effects

Strong evidence was provided on the causal effect between LDL-C and the risk of cardiovascular disease by means of LDL-C concentrations and the cumulative duration of exposure of the vasculature to LDL-C [30]. Consequently, long-term safety and tolerability of lipid-lowering treatments are important to determine their therapeutic compliance and their ability to lower cardiovascular mortality [28].

Different studies have found a satisfactory safety profile for both alirocumab and evolocumab. Symptoms of immune activation, which is often a concern with therapies targeting RNA, were rare in association with inclisiran; there were few instances of flu-like symptoms and no observed elevations in C-reactive protein. Besides some mild adverse events like nasopharyngitis and injection-site reactions, some neurocognitive events were recorded in the monoclonal antibody therapies such as delirium, dementia, cognitive and attention and amnestic disorders, disturbances in thinking and perception, and mental impairment disorders [31]. A previous phase III EBBINGHAUS (Evaluating PCSK9 Binding Antibody Influence on Cognitive Health in High Cardiovascular Risk Subjects) trial evaluating evolocumab versus placebo reported nonsignificant difference [31, 32], yet after increasing the power of the study, contrasting results were reported. However, a doubt regarding these neurocognitive adverse effects has been created and led to a rise in skepticism regarding them.

Fortunately, studies done after those trials erased all doubts that existed about their safety profile. Alirocumab was assessed in the ODYSSEY long-term trial for cardiovascular events and other adverse events in patients on maximally tolerated doses of statins. Findings showed that, compared to placebo, alirocumab caused myalgia reactions at the injection site, in addition to mild neurocognitive and ophthalmologic events. Furthermore, a higher proportion of patients with vitamin E or K levels were lower than the acceptable range in the alirocumab group, yet with no clinically significant difference [33]. Therefore, the administration of alirocumab was not associated with higher rates of neurocognitive adverse effects, even with a low LDL-C.

## 7. Safety of PCSK9 Inhibitors in DM Patients

Almost 72% to 85% of diabetes mellitus (DM) patients suffer simultaneously from dyslipidemia. Therefore, they constitute a special group requiring lipid-modifying therapy [32]. Currently, statins are the main therapy in dyslipidemia management but have been shown to increase the risk of new-onset diabetes, occurring in about 0.2% patients to 9-44% in different studies [34]; to counter this; previous investigations assessed the effect of the inhibitor of PCSK9 on the incidence of DM.

On the metabolism of glucose, different groups of lipid-lowering drugs have different effects. However, several lipid-lowering medications, such as statins, may also raise the chance of developing new-onset diabetes mellitus. However, some other lipid-reducing medications such bile acid sequestrant (BAS) may result in a smaller drop in LDL-C while still improving glucose metabolism and lowering HbA1c [35]. In both the evolocumab and alirocumab trials, patients had a lower cardiovascular risk despite having a history of diabetes. More particularly, neither higher glycated hemoglobin (HbA1c) nor fasting plasma glucose (FPG) concentrations in the evolocumab group and neither a higher risk during follow-up of new-onset DM in patients without DM nor prediabetes at baseline were reported in the FOURIER trial. This trial showed that evolocumab significantly reduced cardiovascular risk in patients with and without DM, making it superior to statins [35, 36]. This was supported by the findings of the ODYSSEY long-term trial, where DM patients in the alirocumab group had constant FPG and HbA1c levels during follow-up (for over 1.5 years) regardless of the presence or absence of mixed dyslipidemia [37]. GLAGOV studies also showed the efficacy of evolocumab combined with statins; progression of atherosclerosis as measured by continuous endovascular ultrasound scans, up to LDL-C levels of 0.5 mmol/L, showed the first signs of sustained benefit. This study was followed by a FOURIER cardiovascular outcome study conducted on almost 27,000 patients with stable ASCVD. Follow-up in OSLER was only 11 months, and in ODYSSEY, it was 78 weeks, which is too short for medications supposed to be used as a life-long treatment. With statins, 16 years and millions of treated patients were included before it was established that they are associated with a significant increase in the incidence of incident type 2 diabetes and myopathy.

Up to date, guidelines are enforced on the importance of the variability of treatment effects in patients as the treatment can be different for a patient regarding many additional factors including age, gender, or race. It is also stressed that treatment with statins, ezetimibe, or PCSK9 inhibitors should occur simultaneously with the promotion of a healthy lifestyle [38]. A previous study reported recommendations from several guidelines on approaches to PCSK9 inhibitors in primary prevention of cardiovascular disease with a consideration of diabetic and nondiabetic patients [39]. Based on a treatment protocol for hyperlipidemia that was adopted from the 2019 AHA/ACC guidelines, the guideline for the primary prevention of cardiovascular disease emphasizes on the use of statin, ezetimibe, and other PCSK9 inhibitors simultaneously with a consideration of patient age and family history and other risk factors [39].

## 8. Safety of PCSK9 Inhibitors on Vitamin E and Steroidogenesis

A large study conducted on 977 diabetic participants treated with evolocumab showed that the treatment was safe even with significantly low LDL-C levels with no adverse events regarding adrenal and gonadal steroid hormone production. Since lipoproteins are eliminated more quickly as a result of evolocumab's normal incorporation of vitamin E into them, various tissues receive vitamin E content as they are cleansed [40].

A phase 3, double-blind, randomized, placebo-controlled DESCARTES study performed on 901 patients with high LDL-C concentrations (≥2.0 mmol/L) receiving either evolocumab or placebo showed a reduction in absolute vitamin E in the evolocumab group by 16%, yet higher by 19% when normalized for cholesterol. Cortisol in evolocumab-treated patients increased slightly from baseline to week 52, but no patient had a cortisol : adrenocorticotropic hormone ratio < 3.0 (nmol/pmol). Among patients receiving evolocumab treatment for 52 weeks, vitamin E levels changed in a way that was similar to lipid changes. Even at very low LDL-C levels, no negative effects were seen in steroid or gonadal hormones [41].

## 9. Cost-Effectiveness and Accessibility of PCSK9 Inhibitors

The cost-effective analysis for any treatment relies on quality-adjusted life year (QALY) and incremental cost-effectiveness ratio (ICER). Specifically, QALY can evaluate the quality of life and life years for each treatment, which in turn compare the efficacies of different treatments, whereas ICER estimates the difference in cost between treatments divided by the difference in their effects, with a smaller ICER indicating better cost-effectiveness. Few evidence was reported on the cost-effectiveness of PCSK9 inhibitors. Their economic value has been assessed by using simulation models with distinct sets of assumptions. Findings comparing PCSK9 inhibitors with statin therapy for secondary prevention showed higher cost-effectiveness ratios from $141,700 to $450,000 per QALY added for the first group, at mid-2018 prices [42]. In addition, in cases with baseline LDL − C levels ≥ 119 mg/dL, lower PCSK9 inhibitor therapy instead of ≥70 mg/dL (≥1.8 mmol/L) resulted in greater cost-effectiveness to $150,000 per QALY added versus $268000. Compared to statins alone, the addition of ezetimibe costs $81,000 per QALY (95% confidence interval (UI), $51,000 to $215,000). The addition of alirocumab costs $308,000 per QALY (UI, $197,000 to $678,000) when compared to statins alone [43].

Studies performed in the US found that the greater impact of evolocumab in reducing cardiovascular risk compared to statins compensated its higher cost. Furthermore, the accumulation of evolocumab in stander treatment (statins) increases the cost-effectiveness of treatment in patients with heterozygous FH, atherosclerotic, CVD, and ASCVD as it is more effective in the reduction of LDL cholesterol level comparing to standard treatment [44]. Another study done in the States from the patient perspective found that for postinsurance approval, only 2.3% were unable to switch or continue therapy given its higher cost [45]. In this study, the financial burden for patients was very reasonable in most cases, with an average monthly out-of-pocket expenditure of $57 and discontinuation rates due to a cost of only 2.3%. This opposes with previous evidence on the high (almost the third) number of patients reported never filling PCSK9i prescriptions primarily due to cost [46].

Contrasting results have been reported on the cost efficacy of PCSK9 inhibitors in the US, with more studies reporting negative outcomes. The high lifetime costs of PCSK9 inhibitors may not be offset by estimated health benefits for most eligible patients. In the model, PCSK9 inhibitors only prove to be cost-effective in secondary prevention for elderly patients with a high absolute risk of CVD. This picture is likely to change as the price falls, whereas they are much cheaper in other countries such as Europe and Norway, suggesting that they can be cost-effective for other regions [47–49].

## 10. Other Therapeutic Interventions

Mipomersen is an innovative medication being considered for the treatment of familial hypercholesterolemia. This second-generation antisense oligonucleotide works by hindering the production of apolipoprotein B-100, leading to a decrease in LDL cholesterol levels. Unfortunately, it has no impact on plasma PCSK9 levels. Although it has the potential to reduce hepatic production of triglyceride-rich very-low-density lipoprotein (VLDL) by targeting apoB-100 mRNA in the liver, it may also result in hepatic steatosis and elevated plasma liver transaminases [50]. A meta-analysis of available data suggests that mipomersen therapy has a higher likelihood of being discontinued, as well as being associated with a higher risk of injection-site reactions, hepatic steatosis, elevated hepatic enzyme levels, and flu-like symptoms [51] (Table 1).

## 11. Conclusion

In summary, predefined analyses of recent randomized controlled trials have shown the best ways to prioritize these therapies to maximize their value in selected high-risk groups. These data are also incorporated into the latest clinical practice guidelines and scientific evidence, expanding the role of PCSK9mAbs compared to previous guidelines. However, although the efficacy and safety of PCSK9 inhibitors have been established by many clinical trials, yet their accessibility and long-term outcomes have not been studied well. The high cost of PCSK9 inhibitors can also affect their purchase later on. Nevertheless, it is suggested that the treatment cost of PCSK9 will also resume with time similarly to statin price a long time ago.

## Figures and Tables

**Table 1:** Comparison of different lipid-lowering agents in terms of mode of action and safety consideration.

Drug class	Agent	Mechanism of action	Safety consideration
Cholesterol absorption inhibitor	Ezetimibe	Blocks the absorption of cholesterol and decreased delivery of intestinal cholesterol to the liver which results in increased cholesterol clearance	Generally well-tolerated with a low incidence of side effects Rare cases of serious allergic reactions have been reported

PCSK9 inhibitor	Alirocumab	Inhibits PCSK9 binding to LDL-Rs resulting in increased cholesterol clearance	Generally well-tolerated with a low incidence of side effects May cause injection-site reactions, such as redness or swelling In some patients, it may increase the risk of neurocognitive events, such as memory loss or confusion
	Evolocumab		

siRNA (small interfering RNA) therapy	Inclisiran	Targets the messenger RNA (mRNA) for PCSK9, reducing the amount of PCSK9 protein available to regulate LDL receptor levels. This results in lowered LDL cholesterol levels	Generally well-tolerated with a low incidence of side effects Injection-site reactions are the most commonly reported side effect May cause liver enzyme elevations, so liver function tests may need to be monitored periodically

Antisense oligonucleotide therapy	Mipomersen	Binds to and inhibits the production of apolipoprotein B-100, a protein that is an essential component of LDL cholesterol. This results in lowered levels of LDL cholesterol in the bloodstream	Can cause injection-site reactions, such as redness, swelling, or pain. May cause liver enzyme elevations and increase the risk of liver damage, so liver function tests may need to be monitored periodically. Can cause flu-like symptoms, such as fever, chills, and fatigue

Vaccines	Anti-PCSK9 vaccines	Stimulates the immune system to produce antibodies that target and neutralize PCSK9, resulting in lower LDL cholesterol levels	—

## Additional Points

*Highlights*. (i) Statins are the first line of treatment for lipid lowering, but a significant number of patients either require additional lipid-lowering therapy for better control of their lipid levels or are unable to tolerate statins. (ii) The most commonly reported side effects of evolocumab and alirocumab are flu-like symptoms, upper respiratory tract infections, and nasopharyngitis. (iii) The most frequently observed adverse reactions of inclisiran include nasopharyngitis, headache, back pain, cough, musculoskeletal pain, and diarrhea. (iv) Their cost-effectiveness has not yet been established clearly, but future outcomes seem promising.
